# Current concepts of photosensitivity in cutaneous lupus erythematosus

**DOI:** 10.3389/fmed.2022.939594

**Published:** 2022-08-25

**Authors:** Benjamin Klein, Manfred Kunz

**Affiliations:** Department of Dermatology, Venereology, and Allergology, University Hospital Leipzig, Leipzig, Germany

**Keywords:** cutaneous lupus erythematosus, UV light, photosensitivity, interferon, DNA damage

## Abstract

Cutaneous lupus erythematosus (CLE) represents a complex autoimmune disease with a broad phenotypic spectrum ranging from acute to chronic destructive cutaneous lesions. Patients with CLE exhibit high photosensitivity and ultraviolet (UV) irradiation can lead to systemic flares in systemic lupus erythematosus. However, the exact mechanisms how UV irradiation enhances cutaneous inflammation in lupus are not fully understood. Recently, new molecular mechanisms of UV-driven immune responses in CLE were identified, offering potential therapeutic approaches. Especially the induction of type I interferons, central cytokines in lupus pathogenesis which are released by various skin cells, have become the focus of current research. In this review, we describe current pathogenic concepts of photosensitivity in lupus erythematosus, including UV-driven activation of intracellular nucleic acid sensors, cellular cytokine production and immune cell activation. Furthermore, we discuss activated pathways contributing to enhanced apoptosis as well as intracellular translocation of autoantigens thereby promoting CLE upon UV light exposure.

## Introduction

The human skin is a complex structure consisting of epidermal keratinocytes, melanocytes, dermal fibroblasts, adipose tissue and numerous immune cells. All these compartments are more or less regularly exposed to ultraviolet (UV) irradiation in many areas of the body surface. In healthy skin, UV irradiation has pleiotropic effects and causes a series of cellular reactions that are both metabolically stimulating, pro-inflammatory or even degenerating (1).

**FIGURE 1:**
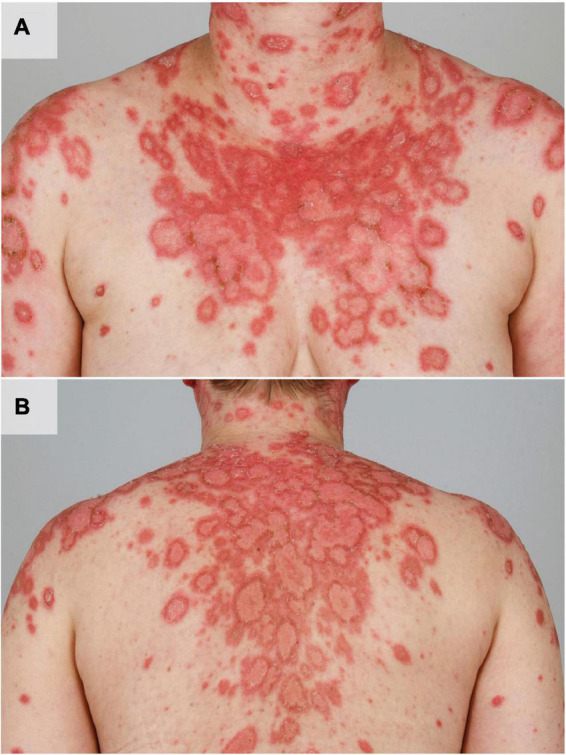
UV induced acute flare of subacute CLE in a female patient with SLE. The patient shows multiple disseminated erythematous, angular and scaly plaques in photodistributed areas such as the shoulders, upper arms, cleavage, neck **(A)** and the back **(B)**.

Cutaneous lupus erythematosus (CLE) represents a chronic recurrent autoimmune disease with multiple clinical manifestations ranging from isolated cutaneous lesions to systemic disease with systemic involvement (2, 3). UV light can cause a deterioration of skin lesions as well as flares of systemic lupus erythematosus (SLE) (Figures 1A,B) (4). Histopathology of CLE lesions shows a perivascular and periadnexal lymphohistiocytic infiltrate and interface dermatitis at the dermo-epidermal border often associated with vacuolization of basal keratinocytes (3). Multiple immune cells are involved in the development of CLE lesions after UV irradiation and lead to further recruitment of the adaptive immune system *via* release of various mediators such as type I interferons (IFN). Recently, activation of type I IFNs after UV irradiation has been linked with activation of *cyclic GMP-AMP-synthase* (cGAS) (5). Upon T cell stimulation, B cells are recruited to the dermal compartment and produce autoantibodies. These autoantibodies are directed in particular against nucleic acids and nucleosome components, contributing to disease progression (3). UV light can also lead to increased antigen presentation and increased apoptosis in lupus erythematosus (LE) (6, 7). The cellular debris that develops after exposure to UV irradiation can accumulate and additionally lead to further immune stimulation (8). In this review, we illustrate the effects of UV light on the development of CLE.

## Effects of ultraviolet irradiation on healthy skin

### DNA damage and cell death

**FIGURE 2:**
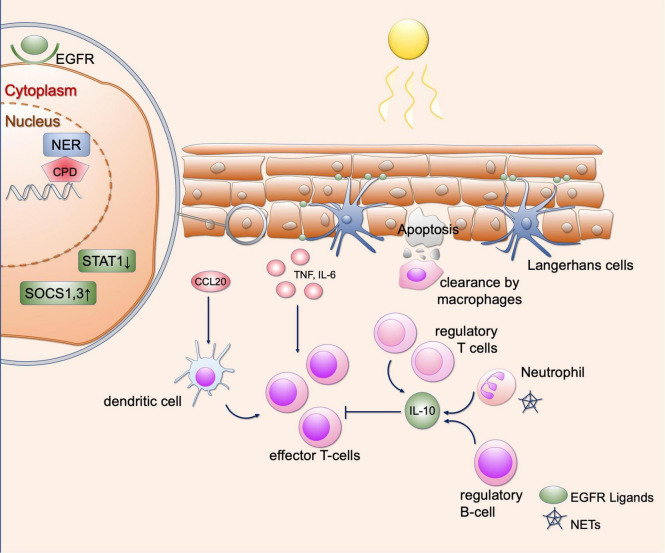
Effects of UV irradiation in healthy skin. Shown are various regulatory mechanisms of the skin that can occur as a result of UV radiation. UV causes DNA damage in keratinocytes, which is repaired by various repair mechanisms (here representing the NER). Furthermore, SOCS1 and SOCS3 as well as STAT1 are downregulated so that the response to cytokines is limited. EGFR ligands provided by Langerhans cells lead to epidermal hyperplasia, which in turn results in protection from further UV radiation. UV radiation further recruits various immune cells to the skin, including regulatory T and B cells and neutrophil granulocytes. These lead to an immunosuppressive environment via the secretion of IL-10. Proinflammatory cytokines and chemokines include CCL20, IL-6, and TNF-α, which can be released by keratinocytes. This causes recruitment of dendritic cells and T cells. In addition, neutrophil granulocytes can undergo NETosis after UV radiation, contributing to immune cell recruitment. This equilibrium of immunosuppressive and proinflammatory responses is necessary to maintain self-antigen tolerance. EGFR, epidermal growth factor receptor; NER, nucleotide excision repair; CPD, cyclobutane pyrimidine dimers; STAT1, signal transducer and activator of transcription; SOCS, suppressor of cytokine signaling; IL, interleukin; CCL20, C-C motif chemokine ligand 20; TNF, tumor necrosis factor; NETs, neutrophil extracellular traps.

Ultraviolet irradiation represents electromagnetic irradiation characterized by shorter wavelengths than visible light (UVA = 400–320 nm; UVB = 320–280 nm; UVC = 280–100 nm). The uppermost boundary layer exposed to UV irradiation represents the epidermis consisting of several layers of keratinocytes, whose complex role in the pathogenesis of skin injury and development of cutaneous diseases has become increasingly appreciated (9). Whereas short wave UVB is not able to penetrate the epidermis, longer UVA waves reach the dermal compartment of the skin and contribute to photoaging *via* reactive oxygen species (ROS). UV irradiation can cause numerous ways of damage in cells such as DNA damage, impairment of DNA repair, changes in gene transcription and induction of cell death, depending on the wavelength and duration of exposure (10, 11). After absorption of UVB irradiation, direct DNA damage occurs resulting in cyclobutane pyrimidine dimers (CPDs) or pyrimidine-pyrimidone photoproducts (6-PPs) (11). CPDs represent cyclic DNA structures of two pyrimidine bases (such as thymine), which are induced in a dose-dependent manner by UV irradiation and contribute to UV-driven apoptosis, which is mediated by activation of the DNA damage response including p53. UV-driven apoptosis is mediated by caspases 9 and 3 *via* ROS and the release of cytochrome C (12–15). Indirect DNA damage occurs upon exposure to UV irradiation of all wavelengths *via* ROS, which can lead to oxidized bases (8-Oxo-2’-deoxyguanosine, 8-oxo-dG) and single-strand breaks (16, 17). As UV enhances the proportion of ROS *via* different mechanisms including alteration of catalase activity, upregulation of nitric oxide synthase and downregulation of protein kinase C, the cell is under oxidative stress (18). Enhanced oxidative stress further leads to altered signaling pathways or, to a lesser extent than CPDs, to apoptosis upon oxidation of different cellular structures such as the outer cell membrane (12). The DNA alterations induced by UV irradiation can either be repaired by different DNA repair mechanisms or result in apoptosis of the cell through blockage of transcription and replication (19). CPDs and 6-PPs are repaired by the nucleotide excision repair (NER) (Figure 2), and oxidative damage is repaired by base excision repair (BER) (12, 19). Once the DNA damage is fully repaired, the cell can continue to perform its original metabolic functions.

### Inflammatory responses after ultraviolet in healthy skin

It is not completely understood how UV radiation influences the transcription of genes and recruitment or activation of immune cells in the skin. – Knowledge of whole-tissue responses to UV irradiation is mainly based on gene transcription studies on skin samples and functional analyses of skin cells. One earlier study evaluated the skin response 24 h after narrowband UVB irradiation – which is used in clinical phototherapy – in skin types II and III (20). A total amount of over 1,500 genes were differentially expressed after UVB irradidation, among which were immune-modulating cytokines, chemokines, leukocyte surface markers and antimicrobial peptides (20).

Ultraviolet irradiation is used as treatment option in a number of chronic-inflammatory cutaneous diseases such as psoriasis and atopic dermatitis, which may be explained by context-dependent effects on cytokine secretion. In keratinocytes, UV irradiation leads to upregulation of *suppressor of cytokine signaling* (SOCS) 1 and 3 expression and downregulation of *signal transducer and activator of transcription* (STAT) 1 expression, which results in reduced responsiveness to IFN-γ (Figure 2) (21, 22). Thereby, UVB narrowband irradiation can downregulate inflammatory cytokines such as *Interleukin* (IL)-12, IL-23 and other IFN-γ-associated genes (23). Inflammatory responses to UV are well-known and can result in extensive sunburn after prolonged exposure. *In vitro* studies with keratinocytes treated with UVB irradiation revealed synthesis of inflammatory cytokines such as *tumor necrosis factor* (TNF)-α, IL-1α, IL-6, IL-8 and IL-10 (Figure 2) (24–27). Furthermore, dermal fibroblasts release TNF-α when exposed to UVB (28). After treatment by UVB irradiation and IL-1α, synergistic effects for TNF-α secretion were observed in fibroblasts and keratinocytes through enhanced gene transcription, suggesting that IL-1α released by surrounding immune cells may enhance the inflammatory response to UV light (29). Furthermore, the production of TNF-α is stimulated in keratinocytes by the damage of non-coding RNA sequences, which are subsequently recognized by *toll-like receptor* (TLR) 3 (30). Thereafter, TNF-α is secreted in a *Toll/IL-1R domain-containing adaptor-inducing IFN-*β (TRIF)-dependent manner (30). After photoprovocation, certain nucleosome subunits as well as small nuclear ribonucleoproteins were upregulated, which are known as autoantigens in SLE (31). This shows that expression of autoantigens also occurs in healthy skin after UV light exposure (31). It should be noted that in the latter mentioned study, all samples were taken from female patients, so that a gender biases in transcriptomic changes after UV cannot be excluded.

With regard to immune cells, it has been shown that UV irradiation can result in an immunosuppressive environment. Neutrophils migrate into the skin after UV exposure and maintain an immunosuppressive environment *via* the secretion of IL-10 (Figure 2) (32). Regulatory B cells may be recruited by IL-10, which can inhibit a dendritic-cell-induced T cell response (Figure 2) (33). However, ROS-dependent induction of human neutrophil extracellular traps (NET) was reported after UV irradiation treatment *in vitro* (34). NETs can activate the immune system *via* intracellular *cyclic GMP-AMP synthase* (cGAS), resulting in type I IFN secretion (35) and thereby promoting a proinflammatory environment.

In T cells, many regulatory mechanisms in the skin after UV irradiation were identified: On the one hand, UV irradiation induces recruitment of CD4^+^, CD25^+^ regulatory T cells, which secrete IL-10 and contribute to an immunosuppressive environment (Figure 2) (36). Moreover, UV irradiation can lead to polarization of skin resident T cells to IL-17 secreting cells *via* extracellular ATP released by keratinocytes: It has been shown that extracellular ATP in increased by UV radiation. After addition of medium of irradiated keratinocytes on T cells, stimulation of IL-17 production was observed (37). This is mediated by ATP, which after binding to P2×7 receptor leads to the release of IL-1 *via* activation of caspase 1. IL-1 further stimulates IL-17 production in T cells (37). Moreover, different subtypes of CD11c + dendritic cells were shown to be present after UV exposure to narrowband-UVB irradiation (20). The authors identified upregulation of certain chemokines such as CCL20 and CCL2, which are able to recruit dendritic cells and T cells into the skin (20). In summary, context-dependent T cell recruitment occurs after UV irradiation, which may result in an immunosuppressive or proinflammatory environment.

Langerhans cells also play a role in mediating the effects of UV light and limit damage to keratinocytes. They express ADAM17, which is induced after UV irradiation (38). This results in secretion of epidermal growth factors receptor (EGFR) ligands by Langerhans cells, further leading to epidermal hyperplasia, which protects against subsequent UV exposure (Figure 2) (38).

As outlined above, the effects of UV light in healthy skin are not exclusively immunosuppressive, but show an interplay of proinflammatory and immunosuppressive cytokines and cell populations. In patients with photosensitivity, as in CLE, the uncontrolled inflammatory response to UV occurs because of numerous mechanisms, which are detailed below.

## Lupus erythematosus – mediators of disease

Lupus erythematosus (LE) is a complex autoimmune disease where a combination of genetic susceptibility and promoting environmental factors results in disease exacerbation (39–41). Multiple cytokines such as type I IFN, IL-6 and TNF-α and chemokines such as CXCL10 are involved in the pathogenesis of LE (3, 39, 42). CLE lesions have a strong type I IFN signature and the level of IFN-induced genes correlates with disease activity of SLE (43, 44). Nucleic acids represent potential ligands for type I interferon release that can be recognized by pattern recognition receptors (45). Accumulation of nucleic acids may be caused by a deficiency of nucleases, some of which have been identified as genetic susceptibility genes in LE (45). In addition to type I IFN, impairment in the opsonization of apoptotic cells have been detected in SLE patients (46). Furthermore, numerous immune cells such as myeloid and plasmacytoid dendritic cells, macrophages and T cells are involved in the pathogenesis of CLE and their functions have been studied after UV radiation (39). In the subsequent sections, mechanisms of photosensitivity in lupus are explained in more detail.

## Effects of ultraviolet irradiation in the development of lupus erythematosus

### Presentation of autoantigens

**FIGURE 3:**
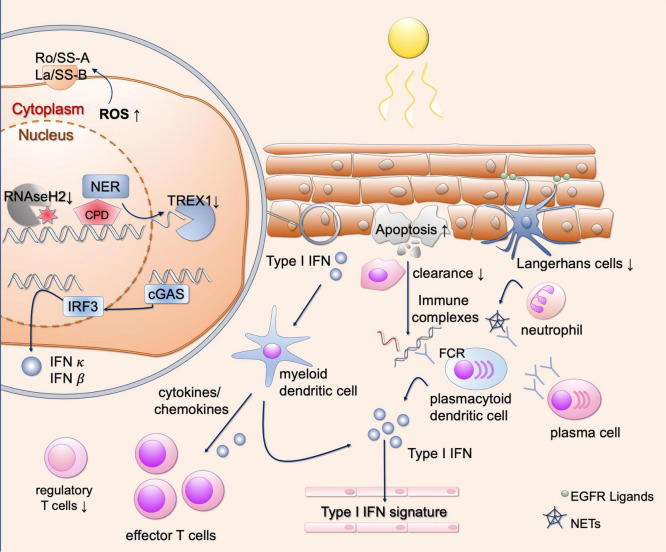
Different mechanisms of photosensitivity in lupus. Upon UV-light, enhanced apoptosis occurs in lupus patients. These apoptotic cells are incompletely engulfed by phagocytes, resulting in secondary necrosis and immune complex formation after binding to autoantibodies. This results in Fc-dependent production of type I IFN, contributing to the interferon signature in blood in lupus patients. Keratinocytes, present autoantigens such as Ro/SSA on the outer surface after UV irradiation. They show DNA damage and enhanced ROS production after UV irradiation, inducing oxidated DNA which is resistant against TREX1-mediated degradation. Free nucleic acids induce cGAS activation and production of type I IFNs. Subsequently, different types of immune cells are recruited to the skin, further activating a strong T cell response. Langerhans cells produce EGFR ligands to protect keratinocytes from UV induced damage, but they are reduced in lupus skin. Neutrophils undergo NETosis after UV irradiation, contributing to the development of immune complexes. EGFR, epidermal growth factor receptor; ROS, reactive oxygen species; NER, nucleotide excision repair; CPD, cyclobutane pyrimidine dimers; TREX1, three prime exonuclease 1; cGAS, cyclic GMP-AMP synthase; IFN, interferon; IRF3, interferon regulatory factor 3; FCR, Fc receptor; NETs, neutrophil extracellular traps.

After exposure to UV irradiation, the localization of certain proteins in the cell changes. These may eventually be recognized as autoantigens as it was shown for the ribonucleoproteins Ro/SS-A and La/SS-B. After UV radiation, these autoantigens are translocated to the outer cell membrane of apoptotic keratinocytes (Figure 3) and can subsequently be bound by antibodies, thus forming immune complexes (6, 47, 48). After exposure to UV radiation, these two antigens become oxidized autoantigens through increased generation of ROS, which in turn increase immunogenicity (49, 50). The influence of TNF-α can also increase the surface expression of Ro52/SS-A and La/SS-B on keratinocytes (51). Expression analysis of Ro52 after UV irradiation showed increase in CLE and other inflammatory skin diseases such as psoriasis, lichen planus and atopic dermatitis (52). Increased expression of Ro52 after UV irradiation may be favored by type I IFN and oxidative stress (53). Furthermore, expression of Ro52 is enhanced by the influence of TNF-α, which itself can also be induced by UV irradiation (54). Evidence to date regarding the translocation of Ro/SSA indicates that this is most likely due to oxidative stress (49). Concerning La/SS-B, recent evidence showed that the translocation from the nucleus to the cytosol is due to redox-dependent conformational changes of La, which can be induced by UV irradiation and is cell-type specific (55). Nevertheless, the exact cellular circumstances under which Ro/SSA and La/SSB presentation at the cell surface arises and how this may be influenced are not fully understood. Ro/SS-A and La/SS-B antibodies are frequently detectable in subacute cutaneous lupus erythematosus (SCLE) and SLE and are also associated with increased photosensitivity (3). These antibodies were initially described in patients with Sjögren’s syndrome, and this patient population also exhibits photosensitivity to some extent (56). This is also described for other antigens such as Sm, RNP and Ku, which are also detectable in SLE (57). Once the autoantigen is bound by antibodies, plasmacytoid dendritic cells as well as B cells can be activated or expanded by the formation of immune complexes (58). Therefore, changing of the cellular localization of autoantigens may cause both increased presentation and increased binding to antibodies.

### Increased apoptosis and reduced clearance of apoptotic cells

In healthy skin, elimination of apoptotic cells that develop after UV radiation is realized by phagocytes to engulf the cell debris by phosphatidylserine-dependent efferocytosis (59). This elimination together with an anti-inflammatory environment, consisting of secreted TGFβ and IL-10, ensures that self-tolerance remains active (Figure 2) (60). Phagocytes recognize membrane components as well as antigens bound by antibodies. The remaining nucleic acids are eliminated by nucleases such as DNase1 (8, 60, 61). However, the capacity of phagocytes is limited and their function of removing apoptotic cells may be impaired, resulting in enhanced amount of autoantigens which can be presented to immune cells (60). In lupus, the number of apoptotic cells measured by TdT-mediated dUTP-biotin nick end labeling (TUNEL)-staining and activated Caspase 3 is already increased in CLE patients without irradiation (7, 62). After UV irradiation, the epidermis of various subtypes of CLE exhibits significantly increased apoptotic cells compared to healthy controls (Figure 3) (7). This higher rate of apoptotic cells persists over time, while healthy controls show reduced numbers of apoptotic cells over time, suggesting a delayed clearance in CLE (7, 8). The number of epidermal apoptotic cells was highest in the UVB irradiated population, suggesting a proapoptotic effect by enhanced CPD formation. In addition, epidermal expression of CD95 was found to be increased, suggesting enhanced extrinsic apoptotic pathway (62).

In SLE patients, a deficit in phagocyte function of macrophages derived from monocytes was demonstrated in earlier reports. *In vitro* macrophages differentiated from SLE patients and incubated in patient serum showed reduced and delayed engulfment of autologous apoptotic components compared to controls (Figure 3) (46). However, an intrinsic abnormality in monocyte derived macrophages from lupus patients was not found, as the phagocytosis capacity between patients and controls was not altered with normal human serum (46). Reduced clearance was associated with lower serum levels of complement components (C1q, C3, and C4) (46). In line with this, increased photosensitivity has been reported in patients with complement deficiency of C4A and C2 (63, 64). Furthermore, CD44, an important signaling molecule of the clearance process, was found to be decreased in SLE phagocytes (65). Due to the decreased uptake of apoptotic material, apoptotic components such as nucleosomes can be presented as antigens and stimulate antibody formation by autoreactive B cells. These antibodies (for example anti-double strand (ds)DNA, anti-nucleosomeal, and anti-histone antibodies) are found in numerous patients with LE (3). Apoptotic cells can, when not engulfed by phagocytes, undergo secondary necrosis (8). Cell material derived from secondary necrosis, may increase immunogenicity *via* post-translational modifications (8).

Another mechanism of cell death represents necroptosis, which was shown to be associated with interface dermatitis, a common histopathologic feature in CLE and other inflammatory skin conditions such as lichen planus (66). Whether UV irradiation can enhance necroptotic cell death in lupus keratinocytes has not been shown so far.

### DNA damage, nucleic acid metabolism and type I interferons

As mentioned above, UV irradiation induces DNA damage in a dose-dependent manner. DNA damage itself may contribute to the pathogenesis of LE, as explained by the example of oxidative DNA damage. Oxidized bases such as 8-oxo-dG represent a consequence of UV-mediated damage through ROS induction (12). This damage is normally eliminated by base excision repair (BER) (19). An initial enzyme of BER represents Oxoguanine glycosylase 1 (OGG1) (19). Interestingly, defects in OGG1 increase IFN expression as well as cutaneous inflammation including alopecia in a murine lupus model (67). In addition, decreased expression of this enzyme has been demonstrated in discoid LE (67). To further understand the role of DNA damage in photosensitivity, it might be helpful to take a look at DNA damage syndromes such as Xeroderma pigmentosum, Bloom syndrome or ataxia telangiectasia. These syndromes are caused by defects in proteins of the DNA repair apparatus, and patients can also exhibit enhanced photosensitivity (68–70). Furthermore, some of these DNA damage syndromes exhibit autoimmune phenotypes such as ataxia telangiectasia, where rheumatoid arthritis and the development of autoantibodies were described (71–73). How DNA damage can lead to secretion of cytokines (in particular type I IFN) has been studied in ataxia telangiectasia. A defect in the ataxia-telangiectasia mutated (ATM) gene results in increased activation of the DNA damage response, mainly in the presence of DNA double-strand breaks, induced by genotoxic stress (71). During the repair of DNA double-strand breaks, single-stranded DNA is initially excised (*end resection*) (19). It was shown that in the case of ATM deficiency, single-stranded DNA accumulates in the cytosol and activates the cytosolic DNA sensor cyclic GMP-AMP synthase (cGAS) (71). Subsequently, signaling of cGAMP *via* interferon regulatory factor (IRF) 3 leads to the release of proinflammatory cytokines (71). The release of type I IFN after DNA damage has been described in different cutaneous DNA damage syndromes (74). Also in lupus, the effects of UV irradiation on type I IFN activation has been investigated.

In healthy murine cells, UV irradiation was able to activate cGAS and the stimulator of interferon genes (STING) and also induce a neutrophil-dependent kidney inflammation (5, 75). The substrate of cGAS activation was not specifically identified in the latter study. But these findings could potentially explain the link between photosensitivity and lupus nephritis. cGAS represents an important mediator and eventually a therapeutic target for photosensitivity (Figure 3). The downstream signaling of this protein will further be discussed below.

Cyclic GMP-AMP synthase represents a cytosolic enzyme and is activated by free cytosolic nucleic acids (76–78). However, under steady state conditions, the nucleus together with the nucleic acids is well-separated from cytosolic cGAS. Furthermore, various intra- and extracellular nucleases prevent endogenous nucleic acids from being recognized by DNA and RNA sensors (79). If defects in nucleases occur or the concentration of cellular nucleic acids increases, recognition of these endogenous nucleic acids may occur. As mentioned above, defects of the extracellular exonuclease DNAse1 as well as the intracellular exonuclease three prime exonuclease 1 (TREX1) have been described in various forms of lupus (Figure 3) (80–82).

After double-stranded DNA or single-stranded DNA with stem loop, is recognized by cGAS, cyclic GMP-AMP (cGAMP) is produced (76). cGAMP can activate STING as a second messenger (Figure 3), which stimulates Tank binding kinase 1 (TBK1) and IRF 3 as well as inhibitor of nuclear factor kappa-B kinase subunit beta (IKKβ) and nuclear factor kappa-light-chain-enhancer of activated B-cells (NF-κB) (83). IRF3 acts as a transcription factor on the expression of type I IFN, which binds in an autocrine and paracrine manner to the IFN-α receptor (IFNAR) (42). Activation of NF-κB results in the release of other proinflammatory cytokines (84). Interestingly, oxidative DNA damage (8-oxo-dG as described above) is resistant to TREX1 degradation, thereby potentiating STING-mediated inflammation (Figure 3) (85). The sources of cytosolic DNA may consist of excised DNA strands during DNA repair, reduction of DNA binding proteins in the nucleus, viral or mitochondrial DNA, micronuclei and senescent cells (86).

It has been shown that in murine cells, type I IFN increases both locally and systemically after exposure to UV irradiation (5). By inhibiting the hydrolysis of cGAMP, the UV-induced release of type I IFN is enhanced (5). Furthermore, UV irradiation-induced IL-1β expression was shown to be cGAS-dependent. However, the production of TNF-α and IL-6 was not dependent on cGAS in murine cells after UV irradiation (5). Increased immune cell infiltrates consisting of neutrophils, monocytes, and γ/δ + T cells were detected after UV irradiation, which infiltrate the skin in a cGAS-dependent manner, in a murine model of skin inflammation. In cGAS-deficient mice, less CCL2 chemokine expression was detected after UV irradiation, which may explain the infiltration of monocytes and T cells in wild type mice (5). cGAS-dependent photosensitivity was recently shown in human TREX1- deficient cells from patients with lupus (87). Here, activation of cGAS with subsequent release of type I IFNs also occurred after UV light (87). The authors revealed enhanced CPD formation after UV irradiation due to TREX1 deficiency. This is caused by enhanced ssDNA fragments within the cell, which are more susceptible for CPD formation after UV irradiation (87). Recently, it has been shown that CPDs are released in extracellular vesicles of keratinocytes in a caspase-dependent manner after UVB-radiation (88). Whether these structures activate STING downstream and if these extracellular vesicles are altered in CLE remains a target of research. A potential source of cGAS activation represent DNA fragments which occur in the cytosol during DNA repair and replication (89). This was recently shown in another study which revealed that during NER small oligonucleotides are released, which are then degraded by TREX1 at the 3′end in HeLa cells (90). Nevertheless, it should be noted that TREX1 deficiency plays a role only in some forms of CLE and the mechanisms of photosensitivity seem to be more complex. Whether exclusively cGAS and STING are induced after UV irradiation in lupus is currently not clear. The involvement of other nucleic acid sensors such as retinoic acid inducible gene 1 (RIG1) in mediating UV-induced inflammation in CLE has not yet been elucidated in detail and needs further investigation.

In SLE, apoptosis-derived membrane vesicles containing dsDNA could be identified as ligands cGAS activation. These were increased in the serum of the patients and contained significantly more dsDNA, which can activate cGAS (91). Whether the UV-induced activation of skin cGAS is also caused by apoptosis-derived membrane vesicles is not fully understood and requires further experiments.

Epidermal derived IFN-κ additionally acts as an important cytokine in the pathogenesis of CLE and SLE and is induced by UV irradiation (Figure 3) (92). IFN-κ drives a proinflammatory response of keratinocytes with recruitment of immune cells to the skin, which may contribute to the cellular infiltrate observed histologically in lupus skin (92). Additionally, IFN-κ enhances signaling of other type I IFNs such as IFN-α, suggesting a priming of human keratinocytes. Specific inhibition of IFN-κ reduces the effects of other type I IFNs, making it a potential therapeutic target in CLE and SLE (92). How exactly UV light stimulates IFN-κ secretion and which signaling pathways are involved, warrants further investigation.

Another risk factor for the development of SLE is a mutation in the ribonuclease RNaseH2 (Figure 3) (93). This enzyme removes ribonucleotides from DNA during replication events (94). It has been shown that a lack of removal of ribonucleotides favors the development of CPDs and leads to an activation of type I IFN after UV radiation (93). It is still unclear whether the CPDs themselves or the following DNA damage response lead to type I IFN activation.

Taken together, several genetic alterations can lead to different forms of LE with increased photosensitivity. The exploration of genetic alterations on a cellular level has unraveled mechanisms of photosensitivity.

### Immune cell recruitment

Besides abnormal cytokine release after exposure to UV irradiation, the production of chemokines plays a substantial role in the recruitment of immune cells in lupus patients. As outlined above, UV irradiation triggers the secretion of proinflammatory cytokines such as TNF-α, IL-1, IL-6 (95). Moreover, after solar simulated irradiation, the UV-induced type I IFN response in the skin elicits the secretion of Th1-associated chemokines such as CXCL9, CXCL10, and CXCL11 in T cells, which contribute to the further recruitment of T cells (Figure 3) (95, 96). The secretion of chemokines such as CCL5 and CCL8 is promoted, resulting in skin inflammation. These cytokines are found to be differentially expressed in CLE (95). Furthermore, CCL27, is upregulated after stimulation by TNF-α and IL-1β, resulting in CCR10-dependent T cell recruitment (95, 97). In line with this, histopathology of lupus lesions specifically shows a T-cell dominated inflammatory cell infiltrate at the dermo-epidermal junction zone. In addition, increased expression of the adhesion molecule ICAM-1 was detected in dermal endothelia after UV irradiation in lupus erythematosus tumidus, CDLE and SCLE, supporting recruitment of immune cells after tissue injury induced by UV radiation (98). UV light is considered to be a trigger of interface dermatitis in the context of photoprovocation. Interestingly, regulatory T cells are decreased in CLE (99). This is mediated by type I IFN-dependent suppression of regulatory T cells and upregulation of effector T cells which are induced by UVB in lupus-prone mice (Figure 3) (100). This indicates that UV irradiation dysregulates the T cell response in CLE. An enhanced effector T cell response also results from the production of cytokines and the presentation of autoantigens, both of which are increased after UV irradiation, as described above.

In mice, monocytes were identified as a source of type I IFN production after UV irradiation (101). This type I IFN production is mediated by colony stimulating factor (CSF)-1, which is produced by keratinocytes, resulting in phagocyte infiltration in the skin and development of CLE-lesions (102). Importantly, infiltration of monocytes after UV irradiation is correlated with type I IFN gene expression, highlighting the role of monocytes in the UV-driven immunopathogenesis of lupus lesions in SLE patients (103). Another source of type I IFN in the development of lupus lesions represent plasmacytoid dendritic cells (pDCs), which are enriched in subtypes of CLE (104). pDCs are able to sense immune complexes containing dsDNA by toll-like receptor (TLR) 9, subsequently leading to type I IFN production (Figure 3) (105). Furthermore, modified mitochondrial DNA, generated after exposure to mitochondrial stress and enhanced ROS production, is able to activate pDCs and induce IFN secretion (106, 107). Oxidized mtDNA is enriched in neutrophil extracellular traps of SLE patients and mitochondrial ROS contributes to lupus-like disease in mice (108). Mitochondrial components such as mtDNA and mtRNA can activate specific cytosolic nucleic acid sensors such as cGAS (mtDNA) and thus contribute to UV-mediated inflammation (109). Currently, it is unclear to what extent UV irradiation contributes to the generation of oxidized mitochondrial nucleic acids in the epidermis and whether their recognition results in increased production of type I IFNs. Hence, mitochondrial stress responses after UV irradiation need further investigation.

Neutrophils appear in UV-irradiated skin and contribute to an immunosuppressive environment through secretion of IL-10. They undergo NETosis after UV irradiation and thereby contribute to immune complex formation (8, 34). Neutrophil activation and subsequent cGAS-dependent neutrophil infiltration of the skin and kidney is IL-17-dependent (75). It is unclear which conditions are necessary for neutrophils to produce IL-10 in a limited manner, paving the way for increased inflammation after UV irradiation.

B cells are also involved in the pathogenesis of LE *via* differentiation to plasma cells and production of autoantibodies (Figure 3) (3). However, the extent to which B cells are directly involved in mechanisms of photosensitivity of lupus patients is poorly understood. Different B cell signatures have been detected in different subtypes of CLE, and it remains intriguing to what extent UV induces or influences this signature (110). Mast cells represent another component of the cutaneous immune system, and their function is particularly important in IgE-mediated diseases (111). However, mast cells also proliferate in lupus lesions after UV irradiation (112). These cells are producers of matrix metalloproteinases (MMPs), which are involved in UV-induced cellular aging (112, 113). Increased levels of MMP-2 and MMP-9 could be detected in lupus patients and also correlated with disease activity (114). However, how exactly MMPs contribute to the inflammatory response in CLE warrants further investigation.

### Perspective

As described above, the various mechanisms that contribute to photosensitivity in LE are manifold. Based on this knowledge, the question arises of how to interfere therapeutically with these mechanism to either prevent or treat this disease. Continuous photoprotection is essential for lupus patients (115). A particular target in LE is the type I IFN pathway and its individual regulating factors. Anifrolumab, an antibody directed against IFNAR, was recently approved for the treatment of SLE and also showed efficacy in cutaneous lesions (116, 117). Furthermore, inhibitors of blood dendritic cell antigen 2 (BDCA2), a C-type lectin, and a number of other compounds are currently tested in in clinical trials and show promise in interfering with the pathogenesis of LE and photosensitivity (117). Currently, a study of the JAK inhibitor tofacitinib is being conducted to investigate photosensitivity in lupus patients before and after therapy (NCT05048238, clinicaltrials.gov). With positive results, a milestone for the therapy of lupus patients with photosensitivity could be created. Since cGAS is a key mediator in the effects of UV light (5, 87), exploration of the mechanisms of the cGAS-STING pathway will lead us to better understanding of photosensitivity in lupus. Recently, R-loop structures have been identified in the context of genomic instability and represent a potential source of cGAS activation (118). In Escherichia coli, R-loop structures occurred significantly higher after UV, highlighting their potential role in UV mediated stress responses (119). Hence, the inhibition of cGAS may also have beneficial effects on photosensitivity. The discovery of human-cGAS-specific small-molecule inhibitors such as G108, G140 and G150 has been recently reported based on high-throughput drug screenings (120). These substances require further clinical testing and may be of benefit for patients suffering from photosensitivity.

Taken together, the pathogenic mechanisms of lupus photosensitivity have been a focus of research in recent years. The major findings of these investigations have not only led to a better understanding of lupus pathogenesis but also to a translation into early clinical trials.

## Author contributions

BK had full access to all of the data in the study and took responsibility for the integrity of the data and the accuracy of the data analysis. Both authors contributed to the article and approved the submitted version.
